# Emergency department hyperoxia is associated with increased mortality in mechanically ventilated patients: a cohort study

**DOI:** 10.1186/s13054-017-1926-4

**Published:** 2018-01-18

**Authors:** David Page, Enyo Ablordeppey, Brian T. Wessman, Nicholas M. Mohr, Stephen Trzeciak, Marin H. Kollef, Brian W. Roberts, Brian M. Fuller

**Affiliations:** 1 Department of Emergency Medicine, Washington University School of Medicine in St. Louis, St. Louis, MO 63110 USA; 2 Department of Anesthesiology, Division of Critical Care Medicine, Washington University School of Medicine in St. Louis, St. Louis, MO 63110 USA; 3 Department of Medicine, Division of Pulmonary and Critical Care Medicine, Washington University School of Medicine in St. Louis, St. Louis, MO 63110 USA; 4 Department of Emergency Medicine, Roy J. and Lucille A. Carver College of Medicine, University of Iowa, 200 Hawkins Drive, 1008 RCP, Iowa City, IA 52242 USA; 5 Department of Anesthesiology, Division of Critical Care Medicine, Roy J. and Lucille A. Carver College of Medicine, University of Iowa, 200 Hawkins Drive, 1008 RCP, Iowa City, IA 52242 USA; 6 Department of Medicine, Division of Critical Care Medicine, Cooper University Hospital, One Cooper Plaza, K152, Camden, NJ 08103 USA; 7 Department of Emergency Medicine, Cooper University Hospital, One Cooper Plaza, K152, Camden, NJ 08103 USA

**Keywords:** Hyperoxia, Mechanical ventilation, Emergency department

## Abstract

**Background:**

Providing supplemental oxygen is fundamental in the management of mechanically ventilated patients. Increasing amounts of data show worse clinical outcomes associated with hyperoxia. However, these previous data in the critically ill have not focused on outcomes associated with brief hyperoxia exposure immediately after endotracheal intubation. Therefore, the objectives of this study were to evaluate the impact of isolated early hyperoxia exposure in the emergency department (ED) on clinical outcomes among mechanically ventilated patients with subsequent normoxia in the intensive care unit (ICU).

**Methods:**

This was an observational cohort study conducted in the ED and ICUs of an academic center in the USA. Mechanically ventilated normoxic (partial pressure of arterial oxygen (P_a_O_2_) 60–120 mm Hg) ICU patients with mechanical ventilation initiated in the ED were studied. The cohort was categorized into three oxygen exposure groups based on P_a_O_2_ values obtained after ED intubation: hypoxia, normoxia, and hyperoxia (defined as P_a_O_2_ < 60 mmHg, P_a_O_2_ 60–120 mm Hg, and P_a_O_2_ > 120 mm Hg, respectively, based on previous literature).

**Results:**

A total of 688 patients were included. ED normoxia occurred in 350 (50.9%) patients, and 300 (43.6%) had exposure to ED hyperoxia. The ED hyperoxia group had a median (IQR) ED P_a_O_2_ of 189 mm Hg (146–249), compared to an ED P_a_O_2_ of 88 mm Hg (76–101) in the normoxia group, *P* < 0.001. Patients with ED hyperoxia had greater hospital mortality (29.7%), when compared to those with normoxia (19.4%) and hypoxia (13.2%). After multivariable logistic regression analysis, ED hyperoxia was an independent predictor of hospital mortality (adjusted OR 1.95 (1.34–2.85)).

**Conclusions:**

ED exposure to hyperoxia is common and associated with increased mortality in mechanically ventilated patients achieving normoxia after admission. This suggests that hyperoxia in the immediate post-intubation period could be particularly injurious, and targeting normoxia from initiation of mechanical ventilation may improve outcome.

**Electronic supplementary material:**

The online version of this article (doi:10.1186/s13054-017-1926-4) contains supplementary material, which is available to authorized users.

## Background

Providing supplemental oxygen is ubiquitous in the management of mechanically ventilated patients. Guidelines for the provision of oxygen give recommendations for target oxygen saturations and for the weaning of oxygen therapy [1]. Despite this, the titration of supplemental oxygen in mechanically ventilated patients is infrequent with resultant hyperoxia being common in the intensive care unit (ICU) [2].

While the deleterious effects of hypoxia are appreciated and actively avoided, hyperoxia is regularly accepted [2–4]. This pendulum swing toward hyperoxia may be associated with harm, as increasing amounts of data show worse clinical outcomes associated with elevated levels of arterial oxygen [5, 6]. Patients suffering from an acute ST-elevation myocardial infarction provided with supplemental oxygen were found to have an increase in recurrent myocardial infarction and arrhythmia and larger myocardial infarct size at 6 months [7]. In mechanically ventilated ICU patients, hyperoxia has been associated with mortality and a decrease in ventilator-free days [8, 9]. In patients resuscitated from cardiac arrest and post-ischemic stroke, hyperoxia has also been linked with worse outcome [10–13]. Finally, patients with traumatic brain injury have increased mortality and worse functional outcomes associated with hyperoxia [14–16]. However, these previous data in the critically ill have focused on outcomes associated with relatively prolonged hyperoxia, with the assessment of hyperoxia exposure during the first 24–72 hours of ICU stay, and up to the entire period of mechanical ventilation [5]. Animal data have shown that the negative consequences associated with hyperoxia can be both time-dependent and dose-dependent, and hyperoxia of only a few hours duration can provoke deleterious changes in inflammation and pulmonary mechanics; yet the effect of a relatively brief exposure to hyperoxia in critically ill patients prior to ICU admission is unknown [17–20].

The emergency department (ED) could be a location that provides both impactful and scalable data to study the effects of initial hyperoxia on outcome: lengths of stay for mechanically ventilated ED patients are long enough for hyperoxia to potentially initiate harm, yet short enough to provide novel data on comparatively brief exposures to hyperoxia. Also, excessive administration of oxygen in the ED is common [21–23]. The objectives of this study were to assess the association between the initial exposure to hyperoxia, immediately after endotracheal intubation in the ED, and clinical outcomes among patients who were subsequently normoxic while in the ICU. We hypothesized that hyperoxia in the ED would be associated with an increase in hospital mortality and increased lengths of stay.

## Methods

### Study design and participants

This was a cohort study, using a database of patients that had mechanical ventilation initiated in the ED at a tertiary academic medical center (September 2009 to March 2016). The database was created as part of a clinical investigation that assessed outcomes associated with the implementation of ED lung-protective mechanical ventilation [24, 25]. All mechanically ventilated ED patients were screened for inclusion. The inclusion criteria were the following: (1) adult patients (age ≥18 years); (2) mechanical ventilation via an endotracheal tube; and (3) normoxia (partial pressure of arterial oxygen (P_a_O_2_) 60–120 mm Hg) on day 1 of ICU admission. The analysis was restricted to those patients with ICU normoxia, given the fact that (1) longer periods of exposure to hyperoxia in the ICU have been studied in the past; (2) this approach allowed us to better isolate a relatively brief hyperoxia exposure (i.e. in the ED) to test its association with outcome; and (3) the association between ED hyperoxia and outcome in mechanically ventilated patients had not been studied previously. Exclusion criteria were as follows: (1) death or discontinuation of mechanical ventilation within 24 hours of intubation; (2) chronic respiratory failure requiring mechanical ventilation; (3) presence of a tracheostomy; (4) transfer to another hospital; and (5) presence of acute respiratory distress syndrome (ARDS) while in the ED (defined by the Berlin criteria) [26]. This study was approved by the institutional review board under waiver of informed consent.

### Procedures

Demographic data, comorbidities, laboratory values, vital signs, illness severity, ED length of stay, and etiology of respiratory failure were collected. Data on treatments provided in the ED included the use of vasopressors and antibiotics and amount of intravenous fluid.

Mechanical ventilator settings provided in the ED were collected, along with gas exchange variables, plateau pressure, static compliance of the respiratory system, and driving pressure. Ventilator settings from the ICU were collected twice daily, for up to 2 weeks.

The definitions of comorbid conditions are provided in Additional file 1. Driving pressure (cm H_2_O) was calculated as plateau pressure minus positive end-expiratory pressure (PEEP). Static compliance (mL/cm H_2_O) of the respiratory system (C_RS_) was calculated as:

$$ {\mathrm{C}}_{\mathrm{RS}}=\mathrm{Tidal}\  \mathrm{volume}/\left(\mathrm{Plateau}\  \mathrm{pressure}\hbox{-} \mathrm{PEEP}\right). $$

The primary outcome was in-hospital mortality. Secondary outcomes were ventilator-free, ICU-free, and hospital-free days. Patients were followed until hospital discharge or death.

### Statistical analysis

Patient characteristics were assessed with descriptive statistics, including mean (standard deviation (SD)), median (interquartile range (IQR)), and frequency distributions. Linear interpolation was used to deal with missing data on lactate values (n = 157 patients). The cohort was a priori categorized into three oxygen exposure groups based on P_a_O_2_ values obtained after intubation (i.e. in the ED, one arterial blood gas per patient). Hypoxia was defined as P_a_O_2_ < 60 mm Hg, normoxia as P_a_O_2_ 60–120 mm Hg, and hyperoxia as P_a_O_2_ > 120 mm Hg. Recognizing that there is no formal definition of hyperoxia, a P_a_O_2_ cutoff value of 120 mm Hg was used as it is congruent with the cutoff value used in other cohort studies that examined ICU hyperoxia exposure in a diverse cohort of mechanically ventilated patients (i.e. analysis not isolated to patients post cardiac arrest or those with stroke or traumatic brain injury) [8, 9, 27]. In a post hoc analysis, the hyperoxia group was further categorized into mild (P_a_O_2_ 121–200 mm Hg), moderate (P_a_O_2_ 201–300 mm Hg), and severe (P_a_O_2_ > 300 mm Hg) hyperoxia subgroups.

To assess predictors for the primary outcome of hospital mortality, categorical characteristics were compared using the chi-square test. Continuous characteristics were compared using analysis of variance (ANOVA) or the Kruskal-Wallis test depending on the distribution of the data. The Bonferroni correction was used to correct for multiple comparisons, and differences in P_a_O_2_ categories were considered statistically significant if *P* was < 0.017. Time (in days) to the primary outcome was assessed with the Kaplan-Meier survival estimate and log-rank test, comparing the normoxic and hyperoxic groups. A second Kaplan-Meier survival estimate was also calculated, which included the hyperoxic subgroups.

To determine independent predictors of mortality, a backward, stepwise, multivariable logistic regression model was used to evaluate death as a function of oxygen exposure group. Clinically relevant variables that were statistically significant in univariate analysis at *P* < 0.05 were candidates for model inclusion. The primary exposure of interest was ED P_a_O_2_. As fraction of inspired oxygen (F_i_O_2_) and PEEP are significantly linked to the exposure and statistically collinear with it, they were not entered into the multivariable model, neither was P_a_O_2_:F_i_O_2_ (expected differences based on the primary exposure group). Variables for inclusion or exclusion from the model were selected in sequential fashion based on the significance level of 0.10 for entry and 0.10 for removal. Normality, statistical interactions, and collinearity (i.e. variance inflation factor) were assessed, and the model used variables that were statistically independent. Model goodness of fit was assessed with the Hosmer-Lemeshow test and by examining residuals. Adjusted odds ratios (OR) and corresponding 95% confidence intervals (CI) are reported for the multivariable model, adjusted for all variables in the model. All tests were two-tailed, and a *P* value <0.05 was considered statistically significant.

## Results

### Study population

**Fig. 1:**
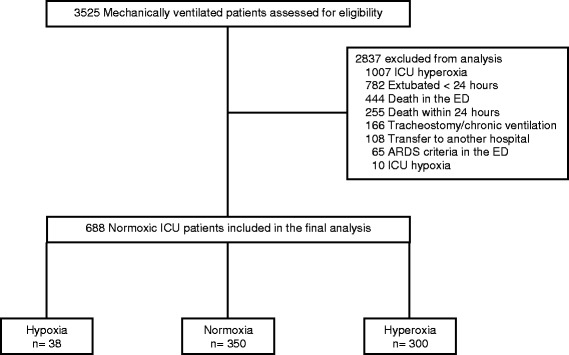
Study flow diagram

The flow diagram of inclusions, exclusions, and the final study population are presented in Fig. 1. A total of 688 patients that were normoxic in the ICU were included in the final analysis.

Table 1 presents baseline characteristics of the study population related to the ED oxygenation group. The median (IQR) ED length of stay (hours) was 5.4 (3.5–7.9), with no difference in ED length of stay between the groups. There were no significant differences between the groups in comorbid conditions or indications for mechanical ventilation. Illness severity, as measured by Acute Physiology and Chronic Health Evaluation II (APACHE II) score, was higher in the ED hypoxia group. The most common reason for initiation of mechanical ventilation was sepsis.

**Table 1:** Baseline characteristics at the time of intubation

	All subjects n = 688	ED hypoxia^a^ n = 38	ED normoxia^b^ n = 350	ED hyperoxia^c^ n = 300	*P* value
Age (years)	59.1 (16.0)	55.0 (16.7)	60.1 (14.7)	58.7 (17.1)	0.024
Female gender, *n* (%)	290 (42.2)	15 (39.5)	166 (47.4)	106 (35.3)	0.015
Race, *n* (%)					
Black	369 (53.6)	23 (60.5)	183 (52.3)	162 (54.0)	0.661
White	317 (46.1)	15 (39.5)	164 (46.9)	134 (44.7)	
Other	7 (1.0)	0 (0)	3 (0.9)	4 (1.3)	
Comorbidities, *n* (%)					
Chronic obstructive pulmonary disease	234 (34.0)	14 (36.8)	122 (34.9)	98 (32.7)	0.383
Malignancy	117 (17.0)	8 (21.1)	57 (16.3)	52 (17.3)	0.655
Congestive heart failure	189 (27.5)	10 (26.3)	92 (26.3)	86 (28.7)	0.893
Diabetes mellitus	252 (36.6)	19 (50.0)	125 (35.7)	107 (35.7)	0.289
End-stage renal disease	52 (7.6)	5 (13.2)	24 (6.9)	23 (7.7)	0.498
Immunosuppression	61 (8.9)	6 (15.8)	33 (9.4)	22 (7.3)	0.287
Cirrhosis	48 (7.0)	1 (2.6)	26 (7.4)	21 (7.0)	0.660
Indication for mechanical ventilation, *n* (%)					
Asthma	18 (2.6)	0	8 (2.3)	10 (3.3)	0.492
Chronic obstructive pulmonary disease	77 (11.2)	4 (10.5)	39 (11.1)	34 (11.3)	
CHF/pulmonary edema	42 (6.1)	5 (13.2)	24 (6.9)	13 (4.3)	
Sepsis	237 (34.4)	17 (44.7)	117 (33.4)	102 (34.0)	
Trauma	119 (17.3)	6 (15.8)	58 (16.6)	53 (17.7)	
Cardiac arrest	42 (6.1)	1 (2.6)	17 (4.9)	24 (8.0)	
Drug overdose	29 (4.2)	1 (2.6)	13 (3.7)	15 (5.0)	
Other	129 (18.9)	4 (10.5)	74 (21.1)	49 (16.3)	
APACHE II score^d^	16 (12–20)	19 (14–25)	16 (12–20)	15 (11–20)	0.011
Systolic blood pressure (mm Hg)	117 (94–145)	105 (75–148)	117 (97–145)	118 (95–144)	0.708
Vasopressor infusion, *n* (%)	178 (25.9)	15 (39.5)	85 (24.3)	77 (25.7)	0.238
Antibiotic administration, *n* (%)	332 (48.3)	23 (60.5)	177 (50.6)	131 (43.7)	0.068
Lactate (mmol/L)	2.3 (1.5–4.2)	3.0 (2.0–5.9)	2.2 (1.4–3.9)	2.3 (1.5–4.2)	0.047
Hemoglobin (g/dL)	12.1 (2.6)	12.1 (2.5)	12.1 (2.6)	12.0 (2.7)	0.335
Intravenous fluids in ED (L)	1.8 (1.8)	1.7 (1.5)	1.7 (1.8)	1.8 (1.8)	0.969
ED LOS (hours)	5.4 (3.5–7.9)	5.9 (3.8–8.4)	5.6 (3.7–8.1)	5.3 (3.5–7.5)	0.985

Continuous variables are reported as mean (standard deviation) and median (interquartile range). *P* values are from the chi-square test for categorical variables, one-way analysis of variance for continuous variables, and the Kruskal-Wallis test (lactate, Acute Physiology and Chronic Health Evaluation (APACHE)). Bonferroni correction: α/*n* of comparisons = 0.05/3 = 0.017. *CHF* congestive heart failure, *LOS* length of stay, *ED* emergency department

^a^Partial pressure of arterial oxygen (PaO_2_) <60 mmHg

^b^PaO_2_ 60–120 mmHg

^c^PaO_2_ > 120 mmHg

^d^Modified score, which excludes the Glasgow Coma Scale

### Oxygenation and mechanical ventilation characteristics

Table 2 shows the ventilator variables in the ED and ICU. There were 300 patients (43.6%) who had exposure to ED hyperoxia, 350 (50.9%) who had exposure to ED normoxia, and 38 (5.5%) who had exposure to ED hypoxia.

**Table 2:** Ventilator variables in the emergency department and day 1 in the intensive care unit

	All subjects n = 688	ED hypoxia^a^ n = 38	ED normoxia^b^ n = 350	ED hyperoxia^c^ n = 300	*P* value
Emergency department					
Tidal volume (mL/kg PBW)	7.5 (6.4–8.6)	6.6 (6.1–7.7)	7.5 (6.4–8.6)	7.6 (6.7–8.7)	0.001
Respiratory rate	16 (14–20)	20 (16–25)	16 (14–20)	16 (14–20)	<0.001
FiO_2_	70 (47–100)	60 (40–80)	68 (40–100)	80 (50–100)	0.004
PEEP	5 (5–7)	8 (5–10)	5 (5–7)	5 (5–5)	<0.001
pH	7.29 (7.21–7.38)	7.26 (7.16–7.33)	7.29 (7.21–7.38)	7.29 (7.21–7.39)	0.354
PaO_2_ (mm Hg)	110 (82–179)	54 (51–55)	88 (76–101)	189 (146–249)	<0.001
PaO_2_/FiO_2_	192 (115–278)	106 (83–141)	129 (92–207)	270 (198–360)	<0.001
Plateau pressure (mmHg)	20 (17–25)	24 (20–28)	20 (17–25)	20 (17–25)	0.006
Static compliance (mL/cm H_2_0)	33 (26–45)	31 (24–36)	33 (25–45)	35 (26–46)	0.071
Driving pressure (cm H_2_O)	14 (11–19)	15 (12–18)	14 (11–19)	14 (11–19)	0.117
Intensive care unit, day 1					
Tidal volume (mL/kg PBW)	8.0 (7.0–8.8)	7.5 (6.5–8.3)	8.0 (7.0–9.0)	7.7 (7.0–8.8)	0.220
FiO_2_	46 (40–60)	46 (40–57)	46 (40–60)	46 (40–60)	0.616
PEEP	5 (5–6)	6 (5–8)	5 (5–6)	5 (5–6)	0.002
pH	7.35 (7.29–7.40)	7.38 (7.30–7.41)	7.35 (7.29–7.39)	7.35 (7.29–7.40)	0.377
PaO_2_ (mm Hg)	97 (82–113)	96 (83–113)	94 (79–109)	100 (84–117)	0.002
PaO_2_/FiO_2_	207 (163–258)	206 (169–244)	200 (156–257)	210 (170–260)	0.053
Plateau pressure (mmHg)	21 (18–25)	22 (20–25)	21 (18–25)	21 (18–25)	0.266
Static compliance (mL/cm H_2_0)	35 (27–43)	33 (27–37)	34 (27–43)	35 (28–43)	0.181
Driving pressure (cm H_2_O)	16 (13–19)	15 (13–18)	16 (13–19)	16 (13–19)	0.342

Continuous variables are reported as median (interquartile range). *P* values are from one-way analysis of variance. Bonferroni correction: α/*n* of comparisons = 0.05/3 = 0.017. *PBW* predicted body weight, *FiO*_*2*_ fraction of inspired oxygen, *PEEP* positive end-expiratory pressure, *PaO*_*2*_ partial pressure of arterial oxygen

^a^Partial pressure of arterial oxygen (PaO_2_) <60 mm Hg

^b^PaO_2_ 60–120 mm Hg

^c^PaO_2_ > 120 mm Hg

Median (IQR) F_i_O_2_ was 80% (50–100) in patients in the ED hyperoxia group, which was significantly higher than the F_i_O_2_ in patients in the ED normoxia (68% (40–100)) and hypoxia (60% (40–80)) groups, *P* = 0.004. In the ED hyperoxia group, there was also a significantly higher median P_a_O_2_ (189 mm Hg (146–249)), and P_a_O_2_:F_i_O_2_ (270 (198–360)), *P* < 0.001.

There were no significant clinical differences between the groups with respect to day 1 ICU oxygenation and mechanical ventilation variables, though some statistical differences existed. Oxygenation and mechanical ventilation variables remained fairly static over the ICU stay, with little difference between day 1 variables and those calculated over the entire duration of mechanical ventilation (Additional file 2: Table S1).

### Clinical outcomes

**Fig. 2:**
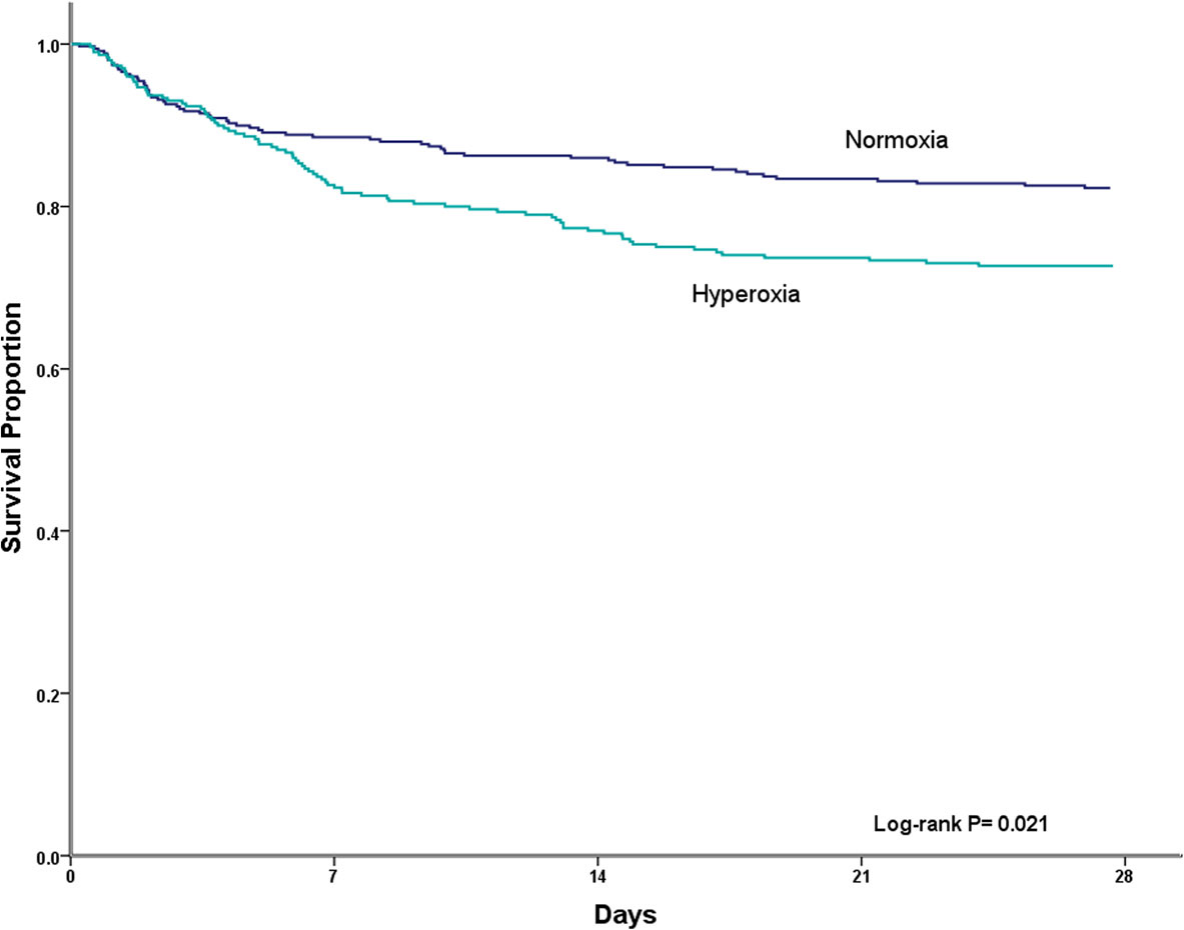
Kaplan-Meier survival curve between the hyperoxia and normoxia groups

In the entire cohort, hospital mortality occurred in 162 (23.5%) patients. Patients with ED hyperoxia had greater hospital mortality (29.7%), when compared to those with normoxia (19.4%) and hypoxia (13.2%). On Kaplan-Meier analysis, survival diverged significantly between the hyperoxia and normoxia groups (log-rank *P* = 0.021, Fig. 2).

The primary outcome analysis is shown in Table 3. The multivariable model was adjusted for age, gender, APACHE II, lactate, ED tidal volume, ED plateau pressure, ICU P_a_O_2_, and oxygen exposure group. After multivariable logistic regression analysis, ED hyperoxia was an independent predictor of hospital mortality (aOR 1.95 (1.34–2.85)). The complete multivariable model results are shown in Additional file 3: Table S2.

**Table 3:** Primary and secondary outcomes according to initial oxygenation group

Outcome	All subjects n = 688	ED hypoxia^a^ n = 38	ED normoxia^b^ n = 350	ED hyperoxia^c^ n = 300	Adjusted odds ratio or between-group difference (95% CI)	*P* value
Primary outcome, *n* (%)						
Mortality	162 (23.5)	5 (13.2)	68 (19.4)	89 (29.7)	1.95 (1.34–2.85)	<0.001
Secondary outcomes (days)						
Ventilator-free	16.5 (10.9)	20.3 (9.7)	17.9 (10.3)	14.2 (11.3)	3.7 (2.0–5.4)	<0.001
ICU-free	15.2 (10.3)	17.9 (9.1)	16.7 (9.8)	13.2 (10.7)	3.5 (1.9–5.1)	<0.001
Hospital-free	10.7 (9.2)	12.8 (9.3)	11.9 (9.0)	8.9 (9.1)	2.9 (1.5–4.3)	<0.001

The *P* value for the primary outcome measure was from the Wald test estimated using a logistic regression model accounting for age, gender, Acute Physiology and Chronic Health Evaluation II score, lactate, emergency department tidal volume, emergency department (ED) plateau pressure, intensive care unit partial pressure of arterial oxygen (P_a_O_2_), and oxygen exposure group. The *P* values for the secondary outcomes are from the independent sample t test, comparing the normoxic and hyperoxic groups

^a^PaO_2_ < 60 mm Hg

^b^PaO_2_ 60–120 mm Hg

^c^PaO_2_ > 120 mm Hg

Secondary outcomes are also presented in Table 3. Compared to the normoxia group, there was a decrease in ventilator-free days (mean difference 3.7; 95% CI 2.0 to 5.4), ICU-free days (mean difference 3.5; 95% CI 1.9 to 5.1), and hospital-free days (mean difference 2.9; 95% CI 1.5 to 4.3) associated with ED hyperoxia, *P* < 0.001 for all.

**Fig. 3:**
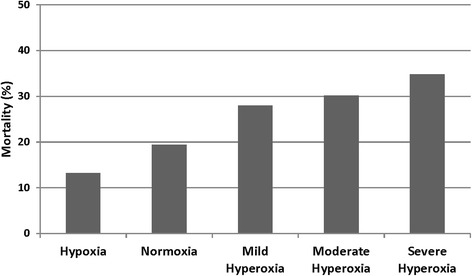
Mortality across oxygenation groups, including hyperoxia subgroups. Hypoxia, partial pressure of arterial oxygen (P_a_O_2_) <60 mm Hg; normoxia, P_a_O_2_ 60–120 mm Hg; mild hyperoxia, P_a_O_2_ 121–200 mm Hg; moderate hyperoxia, P_a_O_2_ 201–300 mm Hg; severe hyperoxia, P_a_O_2_ > 300 mm Hg

The post hoc analysis examined mortality across hyperoxia subgroups. As the level of hyperoxia increased, hospital mortality was greater, (mild hyperoxia 28.0%, moderate hyperoxia 30.2%, severe hyperoxia 34.8%), though this was not statistically significantly different between the hyperoxia subgroups (Fig. 3). On Kaplan-Meier analysis, survival diverged significantly between the normoxia group and the hyperoxia subgroups (log-rank *P* < 0.001, Additional file 4: Figure S1).

## Discussion

### Key findings

This observational cohort study was conducted to examine the association between early hyperoxia exposure and clinical outcomes in mechanically ventilated patients with normoxia during their ICU stay. We found that the liberal use of oxygen in the ED was common, with a median (IQR) F_i_O_2_ of 70% (47–100), and a commensurate pre-ICU hyperoxia rate of 43.6%.

Pre-ICU exposure to hyperoxia in the ED was associated with a mortality rate of 29.7%, higher than in patients in both the hypoxia (13.2%) and normoxia (19.4%) groups. After controlling for confounders, including components of lung-protective ventilation (i.e. tidal volume, plateau pressure) and for baseline imbalances, hyperoxia remained an independent predictor of hospital mortality in multivariable analysis. Additionally, hospital mortality worsened across the hyperoxia subgroups. These data are congruent with prior studies of more prolonged exposure to hyperoxia in the ICU, yet to our knowledge this is the first study demonstrating an association between a comparatively brief early exposure to hyperoxia prior to ICU arrival, and worse clinical outcomes among mechanically ventilated patients [28].

### Relationship to prior work

Clinical guidelines recommend targeting oxygen saturations at 94–98% for most acutely ill patients, and recommend reducing oxygen therapy in patients with satisfactory oxygen saturation [1]. In an observational study of 101 mechanically ventilated patients in the ICU, patients spent > 70% of their total mechanical ventilation time with peripheral arterial oxygen saturation (S_p_O_2_) values of 96–100%, with mean P_a_O_2_ values of 144 mm Hg [29]. In 51 patients mechanically ventilated for > 48 hours, the majority of time was spent with S_p_O_2_ > 98% and 50% of all observations revealed hyperoxia [4]. A Dutch study of 126,778 arterial blood gas measurements from over 5000 mechanically ventilated ICU patients revealed a P_a_O_2_ > 120 mm Hg in 25% of the measurements, yet only 25% of the time was F_i_O_2_ decreased [2]. Finally, data from a single-center and multi-center study has shown that delivery of F_i_O_2_ > 90% is common and little titration of oxygen therapy occurs while patients are mechanically ventilated in the ED [21, 22]. Our current results, along with prior work in this area, further demonstrate that the liberal administration of oxygen is commonplace and hyperoxia is frequently tolerated, and extends these findings into the immediate post-intubation period in the ED.

With respect to clinical outcomes, there has been an increasing amount of data published in this domain over the last decade [6]. These data consist primarily of observational cohort studies with high heterogeneity among them in regards to methods, definition of hyperoxia, and the timing of the assessment of hyperoxia exposure [5, 6]. However, general themes show an association between hyperoxia and harm in the majority of studies. Hyperoxia seems to have a time-dependent and dose-dependent association with outcome, whereby early and severe hyperoxia in the ICU are particularly injurious, and with prolonged exposure [6, 9]. In addition to the production of free radicals, hyperoxia can cause vasoconstriction and a paradoxical decrease in oxygen delivery (within minutes) to prone regional areas (i.e. heart, brain, kidney) [30, 31]. These facts may help explain the findings in the current study, as the hyperoxia observed in the current investigation was in the most immediate post-intubation period, and much more pronounced (P_a_O_2_ 189 mm Hg (146–249) when compared to normoxic patients (P_a_O_2_ 88 mm Hg (76–101)). Additionally, the data suggest that our findings represent an effect of ED hyperoxia (and not carry-over into the ICU), as these patients were normoxic after ICU admission (Table 2), and throughout their ICU stay (Additional file 2: Table S1). This suggests that targeting normoxia from the initiation of mechanical ventilation may improve outcome, and makes complete physiologic sense.

### Significance of study findings

A single-center randomized controlled trial (RCT) recently showed an absolute risk reduction in hospital mortality of 8.2% when normoxia was targeted in ICU patients; this is similar to the 8.3% difference in mortality between the normoxia and hyperoxia groups observed in this study, with survival curves of similar appearance [32]. However, another recent RCT failed to demonstrate any survival signal with a conservative oxygen target in mechanically ventilated patients, though this was a pilot feasibility study not powered for mortality [33]. Our study supports the notion that clinicians liberally administer oxygen in the immediate period of mechanical ventilation and routinely accept (and perhaps even target) hyperoxia. It also supports that this practice may be harmful. While no concrete recommendations can be given on the optimal P_a_O_2_ in mechanically ventilated ED patients, our data suggest little downside in immediately targeting normoxia, and provide data to suggest an opportunity to study this further in the ED.

This investigation has several limitations. It was a single-center study, and the oxygen administration practices and incidence of ED hyperoxia may not be externally valid or apply to other centers. Excess F_i_O_2_ administration in mechanically ventilated patients has been well-documented in the literature, suggesting these data are generalizable outside of our hospital [2, 29]. Given the study design, unmeasured confounders linked to hyperoxia could have accounted for the excess mortality in the hyperoxia group. For example, clinicians may err on the side of hyperoxia intentionally in conditions associated with lower oxygen delivery (i.e. anemia, low cardiac output), greater hypoperfusion, or higher illness severity. However, compared to the normoxia group, hyperoxic patients had similar hemoglobin levels, blood pressure, vasopressor use, lactate, illness severity, and fluid administration. Imbalances in baseline characteristics may have also influenced results, though these were adjusted for in our multivariable analysis. Nevertheless, the current results provide more evidence for the avoidance of excess oxygen, which is not providing any additional therapeutic benefit. While causation cannot be established with the design, the results are consistent with the majority of data on this topic. Furthermore, dose-response suggests causality, and greater mortality was observed across subgroups of increasing ED hyperoxia. The study also reflects real world practice in oxygen management, as it was conducted outside the auspice of a rigidly controlled randomized trial. There is potential for selection bias given the number of excluded patients in the study. The majority of exclusions were due to very early deaths or extubation within 24 hours; it is unlikely that acute hyperoxia would influence outcome in the acutely terminal patient or those stable enough to be extubated within 24 hours. Also, to better isolate an association between ED oxygen exposure and outcome, the analysis was restricted to those patients who were normoxic while in the ICU. Finally, the timing of arterial blood gas analyses was not obtained as part of a formal protocol therefore the exact duration of hyperoxia exposure is unknown. However, we observed marked differences between ED and day 1 ICU oxygenation data, suggesting that the primary exposure was driven by the ED. Furthermore, there were little differences between ICU day 1 data and data calculated over the entire time of mechanical ventilation, suggesting stability in oxygenation data over time (i.e. transient exposures in the ICU were less likely). However, without prospectively following all oxygenation and mechanical ventilation parameters closely in the ICU over time, there still exists the possibility that hyperoxia in the ICU affected some of our results, though our suspicion of this is low. In the future, the exact timing of all mechanical ventilator changes, oxygenation data, and arterial blood gas sampling should be documented to ensure that the exposure (and duration) has been reliably determined.

## Conclusions

ED exposure to hyperoxia is common and associated with increased mortality in mechanically ventilated patients achieving normoxia after admission. This suggests that hyperoxia in the immediate post-intubation period could be particularly injurious, and targeting normoxia at the initiation of mechanical ventilation may improve outcome.

## Additional files

Additional file 1: Definitions of comorbid conditions. (DOCX 13 kb)

Additional file 2: Table S1. Ventilator variables during the entire intensive care unit stay. (DOCX 15 kb)

Additional file 3: Table S2. Multivariable logistic regression model with in-hospital mortality as the dependent variable. (DOCX 15 kb)

Additional file 4: Figure S1. Kaplan-Meier survival curve between the hyperoxia subgroups and the normoxia group. (TIFF 76 kb)

## Abbreviations

ANOVA: One-way analysis of variance
aOR: Adjusted odds ratio
APACHE: Acute Physiology and Chronic Health Evaluation
ARDS: Acute respiratory distress syndrome
CI: Confidence interval
C_RS_: Static compliance of the respiratory system
ED: Emergency department
F_i_O_2_: Fraction of inspired oxygen
ICU: Intensive care unit
IQR: Interquartile range
OR: Odds ratio
P_a_O_2_: Partial pressure of arterial oxygen
PEEP: Positive end-expiratory pressure
RCT: Randomized controlled trial
SD: Standard deviation
SpO_2_: Peripheral arterial oxygen saturation
