# Supplementary material for: Emergency department hyperoxia is associated with increased mortality in mechanically ventilated patients: a cohort study
Source: Crit Care. 2018 Jan 18;22:9. doi: 10.1186/s13054-017-1926-4 (PMC5774130; doi:10.1186/s13054-017-1926-4)
Supplement: Supplementary file 1 — Definitions of comorbid conditions. (DOCX 13 kb) [file 13054_2017_1926_MOESM1_ESM.docx]

**Additional file 1**: Definitions of comorbid conditions

Diabetes Mellitus: Documentation of clinical history in patient’s medical record; current presentation congruent with diabetes mellitus (e.g. diabetic ketoacidosis).

Cirrhosis: Biopsy proven cirrhosis or medical record history suggestive of cirrhosis (ascites, coagulopathy, nodular liver on CT or ultrasound).

Heart failure: Clinical diagnosis on current presentation or history of heart failure in the medical record; includes systolic and diastolic heart failure.

Dialysis/end stage renal disease: Current use of peritoneal dialysis or hemodialysis as an outpatient.

COPD: Not fully reversible airflow limitation; FEV1 <80% + FEV1/FVC <70%; history of COPD in patient’s medical record.

Immunosuppression: Therapy with immunosuppressants, chemotherapy, radiation, long term/recent high dose steroids, active leukemia, lymphoma, or acquired immunodeficiency syndrome (AIDS).

Malignancy: active or chart history of; no requirement for history of, or current radiation/ chemotherapy
