# Supplementary material for: Emergency department hyperoxia is associated with increased mortality in mechanically ventilated patients: a cohort study
Source: Crit Care. 2018 Jan 18;22:9. doi: 10.1186/s13054-017-1926-4 (PMC5774130; doi:10.1186/s13054-017-1926-4)
Supplement: Supplementary file 2 — Ventilator variables during the entire intensive care unit stay. (DOCX 15 kb) [file 13054_2017_1926_MOESM2_ESM.docx]

**Additional file 2, Table S1**: Ventilator variables during the entire intensive care unit stay

|  | **All Subjects**  **n = 688** | **ED Hypoxia^a^**  **n = 38** | **ED Normoxia^b^**  **n = 350** | **ED Hyperoxia^c^**  **n = 300** | ***p* - value** |
| --- | --- | --- | --- | --- | --- |
| **Intensive care unit** |  |  |  |  |  |
| Tidal volume (mL/kg PBW) | 8.0 (7.0 - 9.0) | 7.5 (7.0 - 8.0) | 8.0 (7.0 - 9.0) | 8.0 (7.0 - 9.0) | 0.148 |
| FiO_2_ | 45 (40 - 53) | 45 (40 - 51) | 46 (40 - 54) | 45 (40 - 52) | 0.218 |
| PEEP | 5 (5 - 6) | 6 (5 - 8) | 5 (5 - 6) | 5 (5 - 6) | 0.032 |
| pH | 7.39 (7.34-7.43) | 7.39 (7.35-7.43) | 7.38 (7.34-7.42) | 7.39 (7.35-7.43) | 0.295 |
| PaO_2_ (mmHg) | 99 (85-110) | 93 (84 - 107) | 96 (82-108) | 101 (90-113) | <0.001 |
| PaO_2_/ FiO_2_ | 217 (179-259) | 209 (184 - 237) | 212 (170-253) | 228 (191-265) | 0.011 |
| Plateau pressure (mmHg) | 21 (19 - 25) | 22 (20 - 25) | 21 (18 - 25) | 21 (19 - 25) | 0.105 |
| Static compliance (mL/ cm H_2_0) | 32 (27 - 40) | 32 (27 - 35) | 32 (27 - 42) | 33 (27 - 40) | 0.092 |
| Driving pressure (cm H_2_O) | 16 (13 - 19) | 15 (14 - 18) | 16 (13 - 19) | 16 (13 - 19) | 0.140 |

^a^ PaO_2_ < 60 mmHg; ^b^ PaO_2_ 60-120 mmHg; ^c^ PaO_2_ > 120 mmHg.

PBW: predicted body weight; FiO_2_: fraction of inspired oxygen; PEEP: positive end-expiratory pressure; PaCO_2_: partial pressure of arterial carbon dioxide; PaO_2_: partial pressure of arterial oxygen

Continuous variables are reported as median (interquartile range).

*P* values are from the one-way analysis of variance (ANOVA).
