# Supplementary material for: Emergency department hyperoxia is associated with increased mortality in mechanically ventilated patients: a cohort study
Source: Crit Care. 2018 Jan 18;22:9. doi: 10.1186/s13054-017-1926-4 (PMC5774130; doi:10.1186/s13054-017-1926-4)
Supplement: Supplementary file 3 — Multivariable logistic regression model with in-hospital mortality as the dependent variable. (DOCX 15 kb) [file 13054_2017_1926_MOESM3_ESM.docx]

**Additional file 3, Table S2**: Multivariable logistic regression model with in-hospital mortality as the dependent variable

| **Variables** | **aOR** | **95% CI** | **Standard Error** | ***p* - value** |
| --- | --- | --- | --- | --- |
| Age | 1.04 | 1.03 - 1.05 | 0.01 | <0.001 |
| Lactate | 1.18 | 1.12 - 1.25 | 0.03 | <0.001 |
| ED plateau pressure | 1.03 | 0.99 - 1.06 | 0.02 | 0.092 |
| Hyperoxia exposure | 1.95 | 1.34 - 2.85 | 0.19 | <0.001 |

Removed from model for non-significance: gender (p = 0.79), APACHE II (p = 0.17), ED tidal volume PBW (p = 0.70), ICU PaO2 (p = 0.52), hypoxia exposure (p = 0.99), normoxia exposure (p = 0.16).

ED: emergency department; APACHE: acute physiology and chronic health evaluation; PBW: predicted body weight; aOR: adjusted odds ratio; CI: confidence interval
