# Supplementary figures and images for: Emergency department hyperoxia is associated with increased mortality in mechanically ventilated patients: a cohort study
Source: Crit Care. 2018 Jan 18;22:9. doi: 10.1186/s13054-017-1926-4 (PMC5774130; doi:10.1186/s13054-017-1926-4)

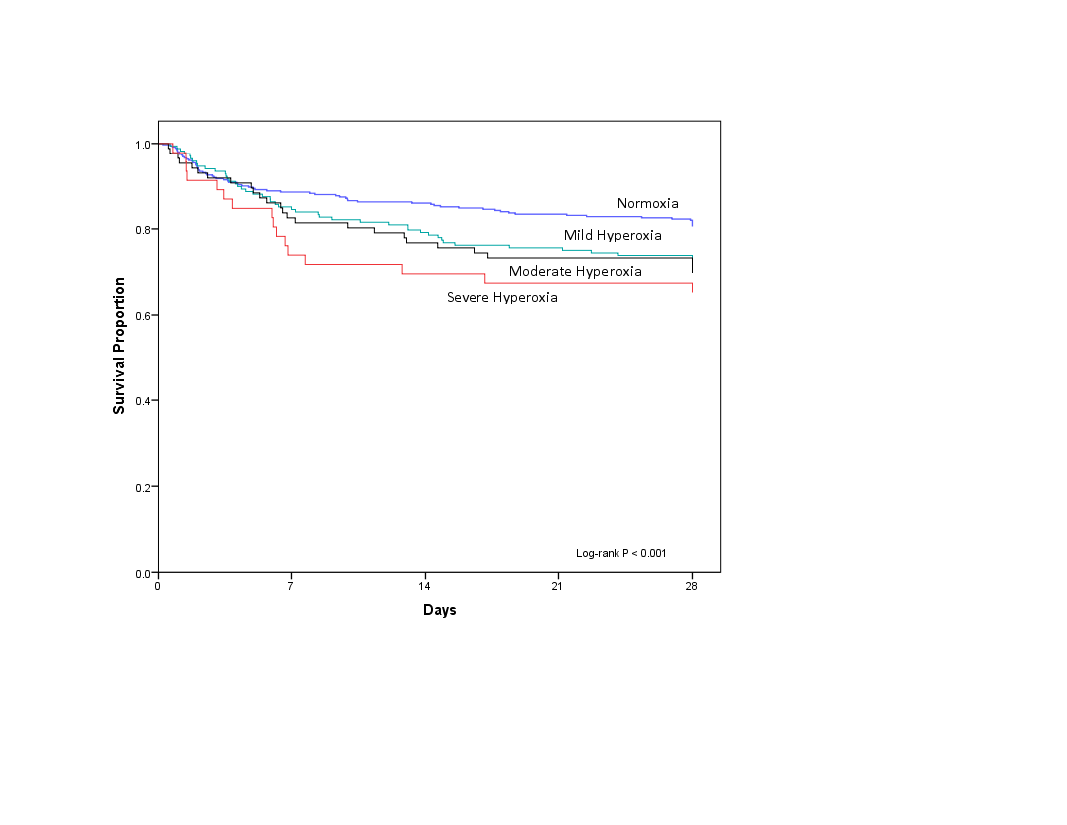

Supplement: Supplementary file 4 — Kaplan-Meier survival curve between the hyperoxia subgroups and the normoxia group. (TIFF 76 kb) [file 13054_2017_1926_MOESM4_ESM.tiff]
